# Influence of different dental scenarios on the accuracy of computerized optical impressions: an in vitro pilot study

**DOI:** 10.1007/s00784-025-06245-0

**Published:** 2025-02-28

**Authors:** Babak Saravi, Clemens L. Paffenholz, Derek Hazard, Ralf J. Kohal, Sebastian B. M. Patzelt

**Affiliations:** 1 Faculty of Medicine, Department of Orthopedics and Trauma Surgery, Medical Center - University of Freiburg, Freiburg, Germany; 2 Private Dental Clinic, Augsburg, Germany; 3 Institute of Medical Biometry and Statistics, Faculty of Medicine and Medical Center, University of Freiburg, Freiburg, Germany; 4 Medical Center – University of Freiburg, Center for Dental Medicine, Department of Prosthetic Dentistry, Faculty of Medicine, Freiburg im Breisgau, Germany; 5 Private Dental Clinic, Zimmern o.R, Germany

**Keywords:** Intraoral scanner, CAD/CAM, Accuracy, Preparation design, Digital impression

## Abstract

**Objectives:**

This in vitro study aimed to determine the influence of various dental scenarios on the accuracy of computerized optical impressions (COIM), specifically focusing on trueness and precision.

**Materials and methods:**

Eight resin casts representing different dental scenarios, including fully dentate upper (UU) and lower jaws (UL), full-arch preparation (FA), single abutment preparations (SA), anterior (AB) and lateral bridge preparations (LB), partial crown preparations (PA), and veneer preparations (VE), were digitally captured using an extraoral reference scanner and an intraoral scanner. The datasets from both scanners were superimposed to calculate and statistically evaluate three-dimensional mean deviations.

**Results:**

The accuracy of maxillary full-arch scans was lowest in the PA scenario (trueness: 34.18 ± 5.46 μm, precision: 36.0 ± 14.69 μm) and LB scenario (trueness: 33.18 ± 7.86 μm, precision: 47.70 ± 14.09 μm). The best accuracy was observed in the SA (trueness: 21.38 ± 1.87 μm, precision: 22.25 ± 4.31 μm) and FA (trueness: 23.75 ± 1.51 μm, precision: 15.26 ± 3.41 μm) scenarios, significantly better than UU (trueness: 29.67 ± 0.79 μm, precision: 29.51 ± 1.17 μm). Soft tissue included in the scans of UU and UL lowered accuracy.

**Conclusions:**

The accuracy of COIM varies significantly with different dental scenarios. Scenarios with extensive edentulous areas and complex preparation designs exhibit lower accuracy.

**Clinical relevance:**

Accurate dental impressions are vital for the proper fit of prosthetic restorations. This study highlights that scenarios with less edentulous areas and simpler preparation designs yield better accuracy. Clinicians should be mindful that large edentulous areas and complex preparations can pose challenges for intraoral scanners, requiring careful scanning strategies to mitigate potential inaccuracies.

## Introduction

Since their introduction in the early 1980s, intraoral scanners (IOS) and the capability for computerized optical impression making (COIM) have become integral to everyday dental practice. The accuracy of an impression is crucial in the subsequent denture fabrication process. For digital impressions, accuracy depends not only on the practitioner’s experience and skill [1, 2], but also on the specific dental scenario of the patient [3–9]. Several studies have investigated different intraoral scanners with respect to specific tooth preparation designs [10–15] or various dental scenarios [3–8], confirming their feasibility [16, 17]. Additionally, intraoral data acquisition has been compared to the scanning of casts and conventional impressions, highlighting its competitiveness [18].

In dental offices, practitioners must decide whether to use digital or conventional methods for acquiring impressions based on the patient’s clinical situation. Directly comparing different dental scenarios can provide valuable insights into the optimal use of digital scans. Given that factors such as blood, saliva, and patient movements influence the accuracy of COIM in vivo [11, 19]. In vitro studies are valuable as they allow for standardized methods, facilitating more reliable comparisons of accuracy between different scenarios. To measure accuracy, both trueness and precision must be determined [20]. Trueness refers to the degree by which the test scan deviates from the reference scan, while precision describes the consistency of deviation values in repeated tests.

The aim of this in vitro study was to determine the influence of different dental scenarios, including fully dentate upper and lower jaws, full-arch preparations, single abutment preparations, anterior and lateral bridge preparations, partial crown preparations, and veneer preparations, on the accuracy of COIM, specifically in terms of trueness and precision.

## Materials and methods

### Reference casts

**Fig. 1:**
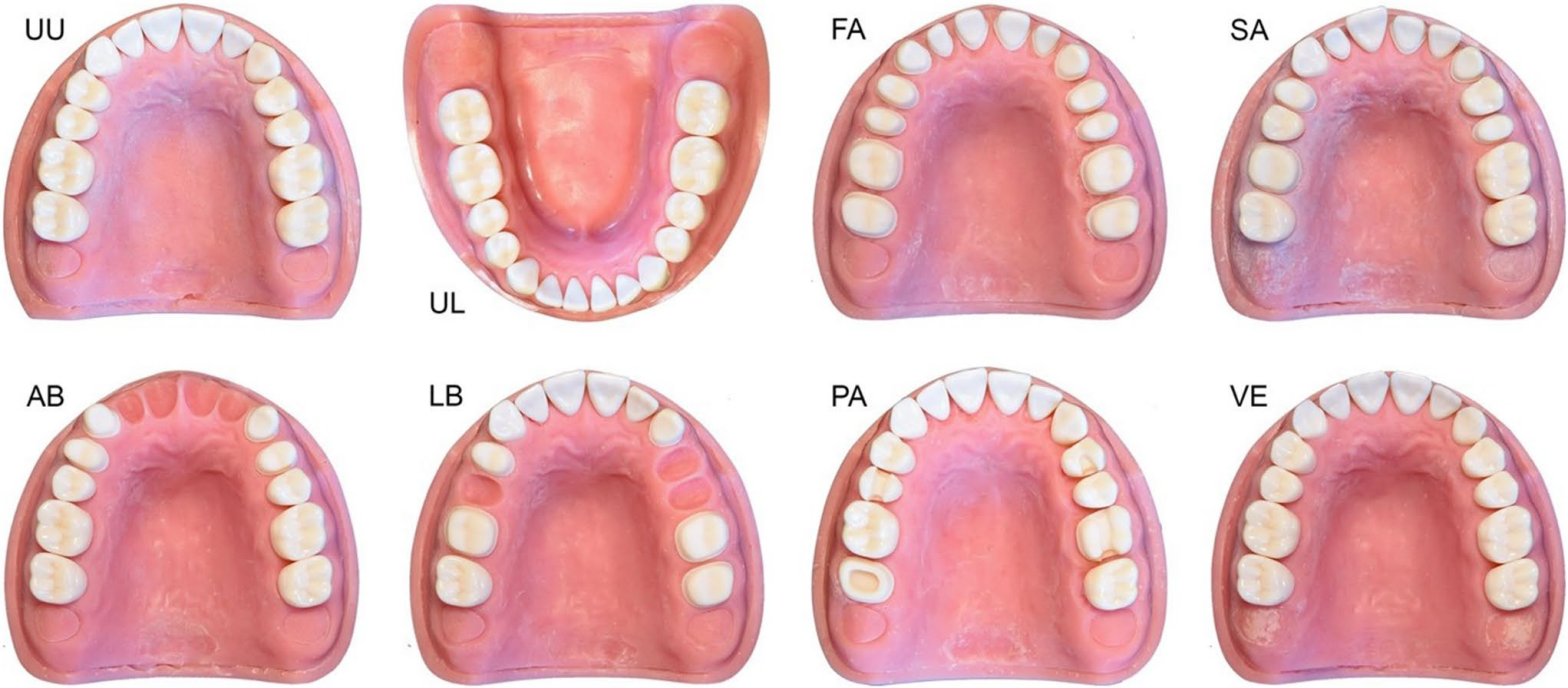
Reference casts (UU: Unprepared upper jaw, UL: Unprepared lower jaw, FA: Full arch preparation, SA: Single abutments preparation, AB: Anterior bridge preparation, LB: Lateral bridge preparation, PA: Partial crown preparation, VE: Veneer preparation)

Dentate maxillary and mandibular study casts (Prosthetic Restoration Jaw Model, Nissin Dental Products Inc., Kyoto, Japan) were replicated using a silicone impression material (Redu-it, Patterson Dental, St. Paul, MN, USA) to create the reference casts. Study teeth (A5A-200, Nissin Dental Products Inc.) were repositioned in the duplicating mold before it was poured with acrylic resin (Selfcuring Denture Acrylic Resin, Lang Dental, Wheeling, IL, USA). For scenarios requiring edentulous areas, the corresponding teeth were removed from the study cast prior to duplication, and the cavities were filled with wax to indicate ovate pontic areas. Various amounts of edentulous areas and different tooth preparations were used to create eight different dental scenarios (Fig. 1): fully dentate upper (UU) and lower jaw (UL) with no tooth preparations, dentate full-arch preparation (FA), single abutment preparation (SA), anterior bridge preparation (AB), lateral bridge preparation (LB), partial tooth preparations (PA), and feathered-edge veneer preparations (VE). For tooth preparation, diamonds with a tapered chamfer and cutting depths of 0.38 mm for anterior teeth and premolars, and 0.54 mm for molars at the crown margin, as well as a bud-shaped diamond for finishing, were used (No. 856P.314.018, No. 856P.314.021, No. 8856P.314.018, No. 8856P.314.021, No. 8368.314.023, Komet Dental/Gebr. Brasseler GmbH & Co.KG, Lemgo, Germany). The full crown preparation angle was six degrees. The PA scenario included an OD inlay on tooth 24, an MOD inlay preparation on tooth 15, a ceramic onlay preparation on tooth 26, and a partial crown preparation on tooth 17, resembling a full crown preparation with an occlusal cut-out.

### Reference scan and test scan

**Fig. 2:**
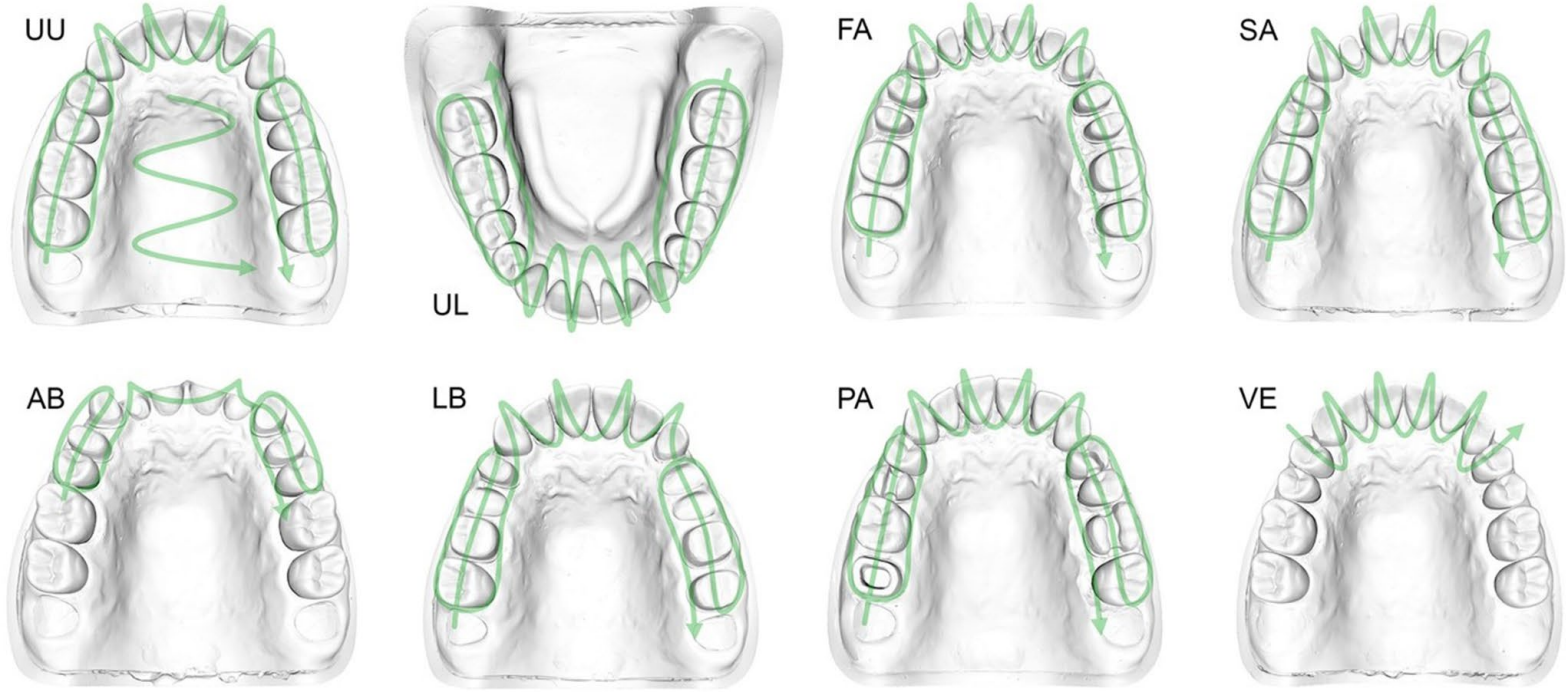
Individual scan path for each reference cast

First, an industrial scanner (ATOS III Triple Scan, GOM GmbH, Braunschweig, Germany) was used to scan all different dental model scenarios once to create the reference data. Then, each dental cast scenario was scanned five times using an intraoral scanner (IOS) (CS 3600, Acquisition software version 3.0.1, Carestream Health, Rochester, NY, USA). All scans with the IOS were performed by the same operator (CLP) after 15 practice scans, under similar ambient conditions (room temperature 23.5 ± 0.5 °C, relative humidity 38 ± 3%), and following a scenario-dependent scan path (Fig. 2). The scan path started in the posterior first quadrant of the upper jaw and the third quadrant of the lower jaw for all scenarios. The occlusal plane of the posterior teeth was first scanned up to the canine, followed by moving the scanner back on the buccal surface of the teeth and then forth on the palatal or lingual sides. In the anterior region, the scanner was moved in serpentine lines over the teeth. Finally, in the second and fourth quadrants, the occlusal surface was scanned first from anterior to posterior, then back on the buccal surface, and forth on the oral surface of the teeth. In the UU scenario, the palate was scanned in serpentine lines from anterior to posterior afterwards. The executed scan path was performed according to the manufacturer’s recommendations shown before starting the scan interface (Acquisition software version 3.0.1, Carestream Health). Insufficiently scanned areas were marked by the software with yellow highlights and green arrows, suggesting the scan direction for correction. Based on these suggestions, these areas were rescanned afterwards. The datasets from both scanners were saved in the Standard Tessellation Language (STL) file format after the scanning processes.

### Data evaluation and 3D comparison

The STL files were imported into a 3D evaluation software (Geomagic Control 2014, 3D Systems, Rock Hill, SC, USA) and trimmed to the specific areas to be analyzed. To ensure high comparability, trimming was done consistently across all scenarios. Orientation for trimming was based on the preparation margin, gingival margin, or the outer edge of the ovate pontic areas on the edentulous areas. For comparing the scanned soft tissue in the UU and UL group, trimming was performed at the deepest point of the mucosal envelope fold buccally and lingually, and along the palatal vibrating line.

From each test and reference scan, the following areas were generated for analysis: the full arch, each prepared tooth isolated for separate evaluation, areas with and without adjacent unprepared teeth for each preparation (SA, PA), and areas of the dental arch with and without soft tissue for UU and UL. In the FA and SA scenarios, the soft tissue areas were trimmed. For AB and LB, each bridge with preparations, ovate pontic areas, and adjacent teeth were set into separate areas. Additionally, areas with only the preparations of each bridge were created. All datasets were superimposed using the best-fit algorithm method of the comparison software. The evaluation involved calculating the deviations in three dimensions (x, y, and z) between the IOS datasets and the reference dataset. For each scan, the mean deviation in the x, y, and z axes was obtained, resulting in both positive and negative deviation values. These deviations were then converted to absolute values for simplification.

For trueness, the mean deviation values (positive and negative) for each scan were considered. With five scans per scenario, this resulted in ten absolute mean deviation values (five positive and five negative) per scenario for statistical comparisons. For precision, the IOS scans of each scenario were compared with each other. Each scenario involved comparing the five scans against each other to evaluate consistency. The choice of five scans per scenario aligns with the methodology of prior studies in similar contexts, ensuring sufficient data for reliable statistical comparisons while maintaining feasibility for a pilot study [3, 5]. To substantiate this choice, we conducted a power analysis. For pairwise comparisons using T-tests, an assumed large effect size (Cohen’s d = 0.8), a significance level of 0.05, and a desired power of 0.8 indicated that five scans per scenario were sufficient to detect significant differences. Furthermore, the linear mixed regression models employed in the study treated individual scans as random effects, allowing robust analysis of within-scenario variability. With 5 scans per scenario across 8 scenarios (total *n* = 40), the sample size ensured adequate statistical power and reliable model estimation. This approach strikes a balance between practical constraints and statistical validity, making it suitable for the exploratory nature of this pilot study. Comparable studies have similarly demonstrated that a sample size of five scans per scenario is sufficient for assessing intraoral scanner performance, further supporting our methodology [3, 5]. This comparison involved calculating the mean deviation (x, y, z), resulting in positive and negative deviation values, which were then converted to absolute values. Each scan provided two absolute mean deviation values. With five scans per scenario, this resulted in ten absolute mean deviation values per scenario for precision. The standard deviation for each comparison was also calculated to assess the consistency of the deviations, providing insight into the variation or dispersion of the deviation values.

The distinction between positive and negative deviations was made to assess the directional tendencies of the errors, offering insight into whether the scanner predominantly overestimated or underestimated specific dimensions. This analysis allows for the identification of systematic biases that may be inherent to the scanning process. However, for statistical comparisons and trueness evaluations, the deviations were subsequently converted to absolute values to focus on the overall magnitude of the errors, ensuring consistency and comparability across scenarios. This dual approach enhances the interpretability of the data while maintaining methodological rigor.

Visual tools, such as color-coded deviation maps, were used to analyze potential deviation similarities across different datasets. The parameters for the 3D comparison were defined as follows: 48 color segments with maximum (dark red) and minimum (dark blue) critical display values of 100 μm and − 100 μm, respectively. This scaling was maintained for full arches and edentulous jaws, with deviation values from 100 μm to 600 μm shown in dark red and from − 100 μm to -600 μm shown in dark blue. Deviations beyond this range were displayed in dark gray, while a deviation of 0.0 μm was shown in light green.

### Statistical analyses

The metrics analyzed included the mean deviation from the IOS compared to the reference scan, reflected as trueness, and the comparison of the scans within each scenario to reflect precision. This overall evaluation aimed to determine the accuracy of the IOS. For statistical analyses, the mean ± standard deviation was computed for each of the metrics and scenarios. Box plots were used for graphical presentations. Pairwise comparisons were performed using T-tests. Linear mixed regression models, with each scan of the specific scenario as a random effect, were used to assess differences among the areas regarding the mean deviation outcomes. A level of statistical significance was set at *p* < 0.05. Due to the exploratory nature of the analyses, p-values and 95% confidence intervals were not corrected for multiple comparisons. Therefore, inferences drawn from them may not be reproducible. All calculations were performed using the statistical software STATA 15.1 (StataCorp LLC, College Station, TX, USA).

## Results

### Trueness

**Fig. 3:**
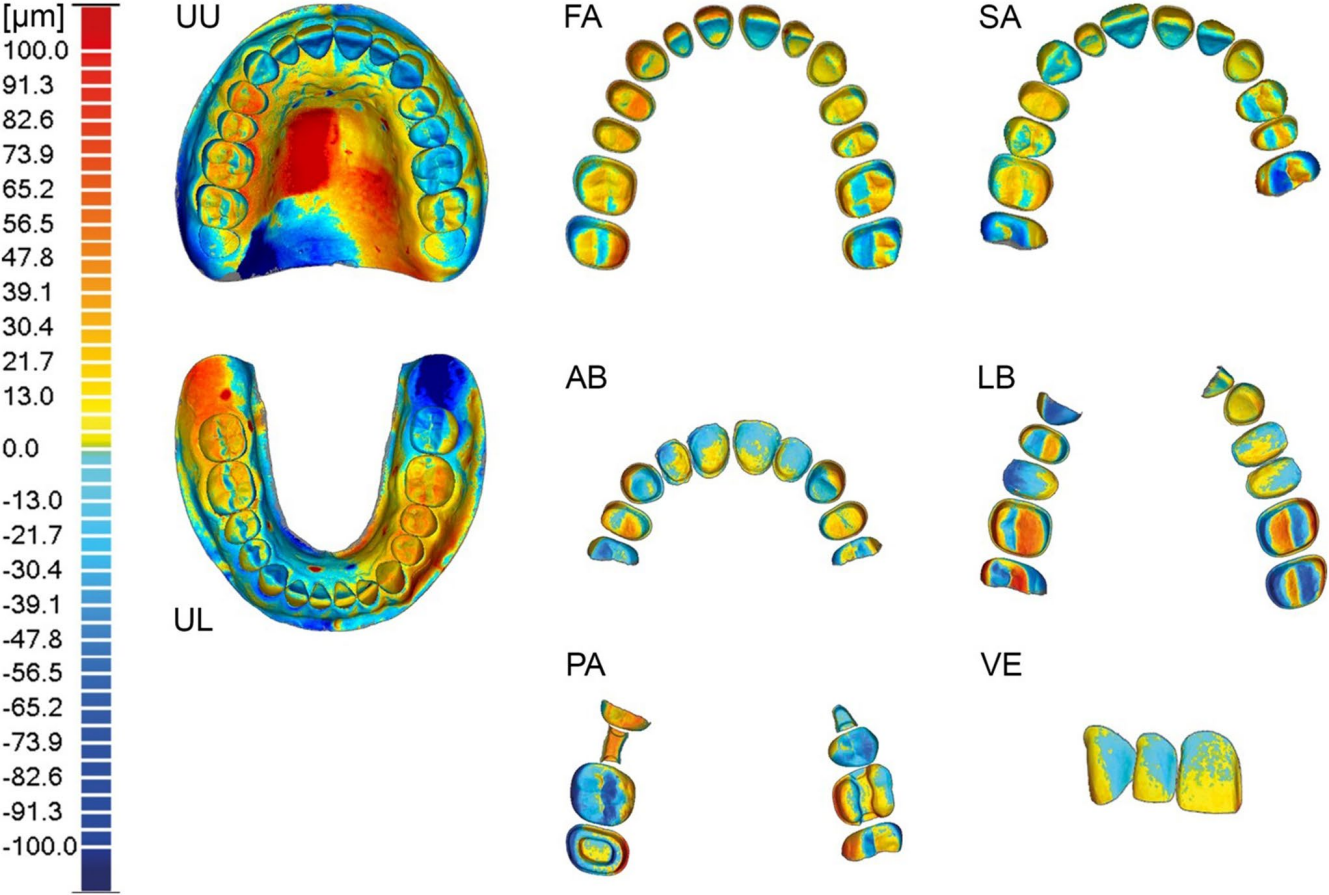
Mean deviations (trueness) of color-coded superimposed datasets (representative example comparison) for each dental scenario (UU: Unprepared upper jaw, UL: Unprepared lower jaw, FA: Full arch preparation, SA: Single abutments preparation, AB: Anterior bridge preparation, LB: Lateral bridge preparation, **PA**: Partial crown preparation, **VE**: Veneer preparation)

When comparing the mean deviations (Table 1) of the unprepared scenarios (UU, UL) in terms of trueness, the mean deviation values were higher when the soft tissue was included in the area of analysis, indicating lower accuracy. High deviations (≥ 100 μm) between the reference scanner and the IOS were observed in the steep anterior palatal region (Fig. 3). There were no significant differences in mean deviation when comparing the dental arches of the maxilla and mandible (0.84 μm, *p* = 0.5604), suggesting that both arch scans achieved similar trueness. However, when soft tissue was included in both scenarios, the difference became statistically significant (4.08 μm, *p* = 0.0192), reflecting better trueness for the lower jaw.

The best trueness values were achieved in the fully prepared dental arch (FA), anterior bridge (AB) and with the single abutments (SA). In the latter case, the overall trueness of the preparations with and without adjacent teeth were comparable (0.41 μm, *p* = 0.6856). In detail, a significant deterioration of the deviation values of 19.39 μm (*p* = 0.0000) was observed for the left maxillary central incisor when the proximal neighbouring tooth surface was added. Further preparations in SA showed smaller but still significant increases in mean deviation when the adjacent, unprepared teeth were included.

When all preparations of FA were compared to each other with regards to trueness, significant differences were seen (trueness: *p* = 0.0027). However, no deviation tendency in relation to a specific region on the dental arch could be visually detected. When all preparations of SA were compared to each other, no significant differences occurred for trueness (*p* = 0.3152).

The lateral bridge scenarios (LB) showed a mean deviation of 16.32 ± 1.38 μm in the first quadrant (one missing tooth) and 19.04 ± 5.47 μm in the second quadrant (two missing teeth) when proximal adjacent tooth surfaces and ovate pontic areas of the missing teeth were included in the area of analysis. There was a statistically significant difference for trueness between these areas (2.72 μm, *p* = 0.0258) as well as when only the preparations of both quadrants were compared (5.11 μm, *p* = 0.0053). In comparison, for the anterior bridge (AB) with four missing teeth, significant deviations existed in the first quadrant both with (4.66 μm, *p* = 0.0003) and without adjacent teeth and ovate pontic areas (8.80 μm, *p* = 0.0001), but not in the second quadrant in either case (with adjacent teeth: 1.94 μm, *p* = 0.2399, without adjacent teeth: 3.69 μm, *p* = 0.2321). Regarding the partial crown preparations (PA), there was a statistically significant difference between the deviation values of all preparations with and without adjacent tooth surfaces (5.89 μm, *p* = 0.0129).

**Table 1:** Mean deviations (trueness) in Μm, *range: minimum mean deviation (region) - maximum mean deviation (region). Tooth numbering follows the fédération dentaire internationale (FDI) system. (**UU**: unprepared upper jaw, **UL**: unprepared lower jaw, **FA**: full arch preparation, SA: single abutments preparation, **AB**: anterior Bridge preparation, **LB**: lateral Bridge preparation, **PA**: partial crown preparation, **VE**: veneer preparation)

Scan	Description	Dental arch mean ± sd	All preparations mean ± sd	Single preparations (range*) mean ± sd
**UU**	unprepared upper jaw with soft tissue	34.99 ± 5.35		
	- without soft tissue	29.67 ± 0.79		
**UL**	unprepared lower jaw with soft tissue	30.91 ± 3.60		
	- without soft tissue	28.92 ± 1.83		
**FA**	full arch	23.75 ± 1.51	23.75 ± 1.51	11.76 ± 1.34 (16) – 15.72 ± 1.28 (11)
**SA**	single abutments	21.38 ± 1.87	21.79 ± 2.30	12.68 ± 3.01 (23) – 15.35 ± 3.62 (14)
**AB**	anterior bridge	20.98 ± 2.80	21.96 ± 5.67	12.35 ± 1.34 (13) – 14.02 ± 1.32 (14)
**LB**	lateral bridges	33.18 ± 7.86	33.51 ± 10.35	12.17 ± 1.22 (16) – 17.00 ± 5.48 (26)
	− 1st quadrant		13.16 ± 1.00	12.17 ± 1.22 (16) – 14.15 ± 1.74 (14)
	− 2nd quadrant		18.27 ± 7.85	12.55 ± 3.95 (27) – 17.00 ± 5.48 (26)
**PA**	partials	34.18 ± 5.46	28.29 ± 6.52	12.84 ± 0.76 (26) – 19.53 ± 4.48 (24)
**VE**	veneers		17.92 ± 2.32	11,54 ± 0,91 (11) – 14.94 ± 1.39 (13)

**Fig. 4:**
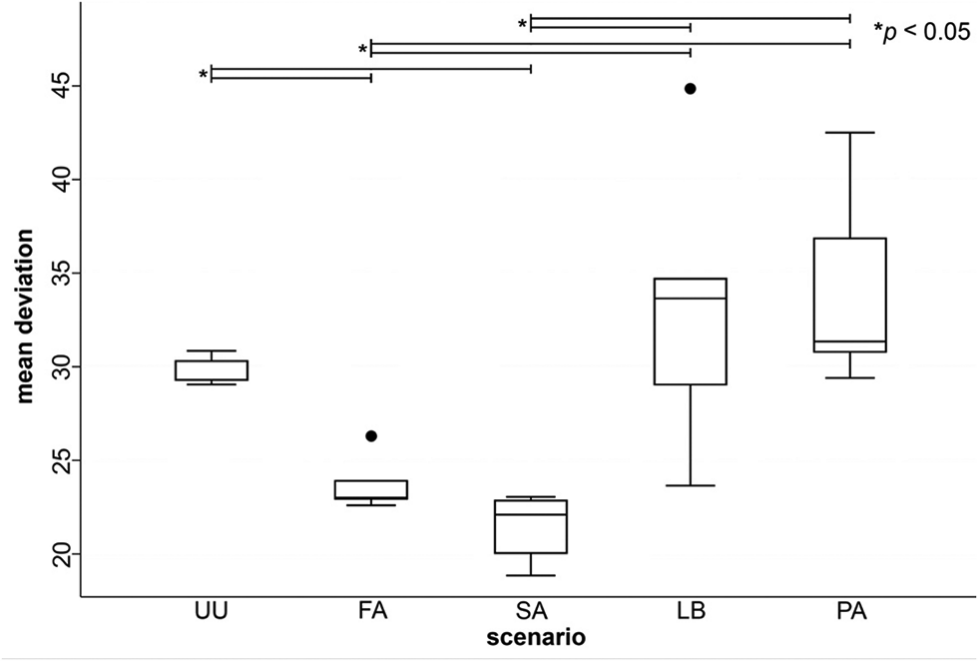
Mean deviations (trueness) of maxillary arches including soft tissue. Statistically significant differences between scenarios marked with asterisks. (UU: Unprepared upper jaw, UL: Unprepared lower jaw, FA: Full arch, SA: Single abutments, AB: Anterior bridge, LB: Lateral bridges, PA: Partials, VE: Veneers)

The mean deviations (trueness) of maxillary arches including soft tissue are shown in Fig. 4. The comparisons between different scenarios revealed several significant findings. Significant differences in trueness were observed between the unprepared upper jaw (UU) and the fully prepared dental arches (FA and SA) (*p* = 0.0000 for both), indicating that the full arch and single abutment preparations achieved better trueness compared to the unprepared upper jaw. However, the difference between UU and the lateral bridge (LB) was not statistically significant (*p* = 0.0839), nor was the difference between UU and the partial preparations (PA) (*p* = 0.0543). The full arch (FA) had significantly better trueness compared to the lateral bridges (LB) (*p* = 0.0000) and the partial preparations (PA) (*p* = 0.0000). Additionally, the single abutments (SA) demonstrated significantly better trueness compared to the lateral bridges (LB) (*p* = 0.0000) and the partial preparations (PA) (*p* = 0.0000). Notably, the comparison between FA and SA did not reveal a significant difference in trueness (*p* = 0.0540), suggesting that both preparation types achieve similar levels of accuracy. Additionally, no significant difference in trueness was found between LB and PA (*p* = 0.7033), indicating that these scenarios had comparable trueness. (Fig. 4; Table 2).

**Table 2:** Overall differences (µm) and p-values of comparisons of maxillary arches including soft tissue regarding mean deviation (trueness) (**UU**: unprepared upper jaw, **UL**: unprepared lower jaw, **FA**: full arch, **SA**: single abutments, **AB**: anterior Bridge, **LB**: lateral bridges, **PA**: partials, **VE**: Veneers)

	Trueness	
	Overall difference (µm)	*p*-value
**UU** vs. **FA**	6.01	0.0000
**UU** vs. **SA**	8.38	0.0000
**UU** vs. **LB**	3.42	0.0839
**UU** vs. **PA**	4.42	0.0543
**FA** vs. **SA**	2.37	0.0540
**FA** vs. **LB**	9.43	0.0000
**FA** vs. **PA**	10.43	0.0000
**SA** vs. **LB**	11.80	0.0000
**SA** vs. **PA**	12.80	0.0000
**LB** vs. **PA**	1.00	0.7033

### Precision

**Fig. 5:**
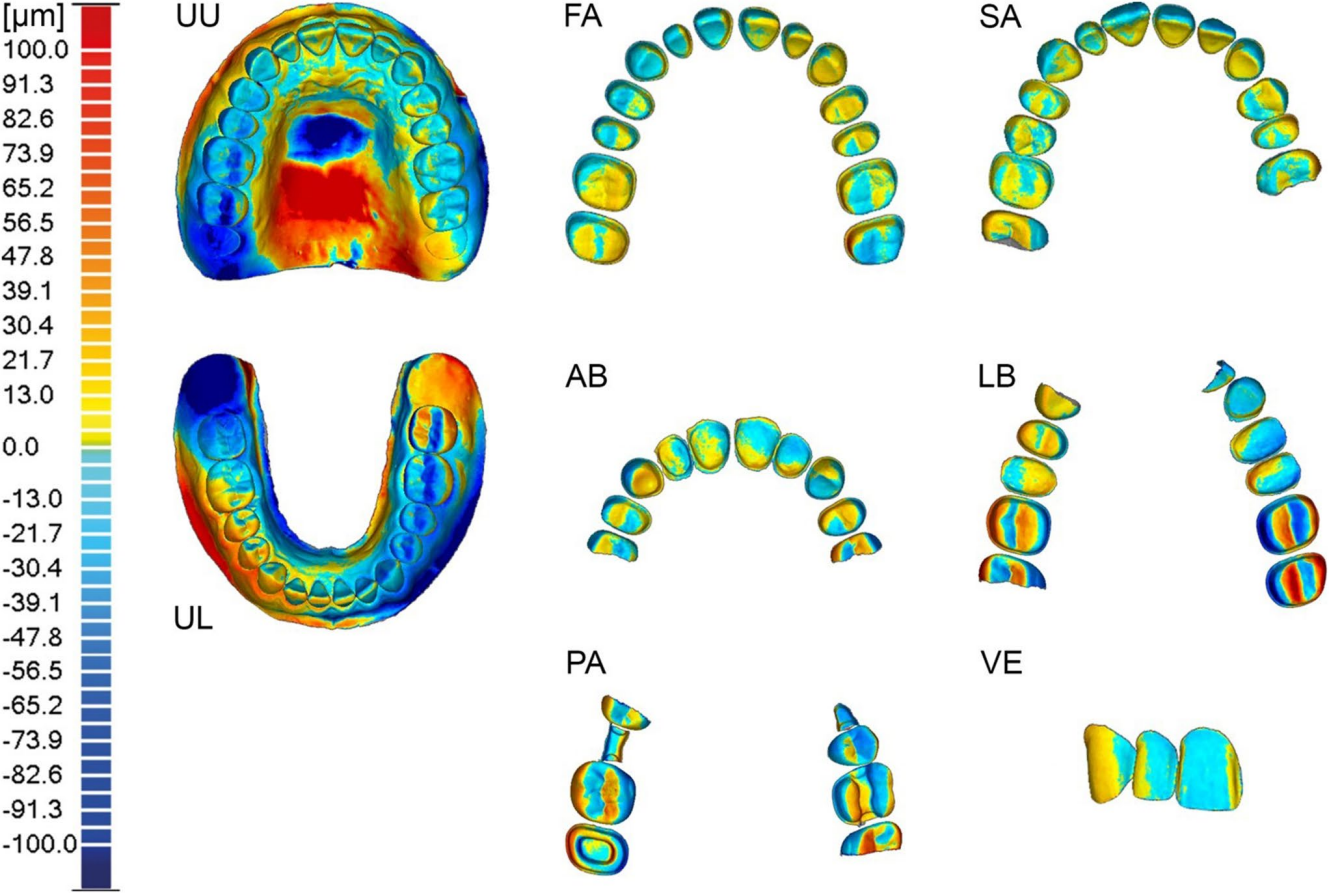
Mean deviations (precision) of color-coded superimposed datasets (one out of five comparisons) for each dental scenario (UU: Unprepared upper jaw, UL: Unprepared lower jaw, FA: Full arch preparation, SA: Single abutments preparation, AB: Anterior bridge preparation, LB: Lateral bridge preparation, PA: Partial crown preparation, VE: Veneer preparation)

Regarding mean deviations (precision), the dental arches without soft tissue differed significantly between the arches (9.68 μm, *p* = 0.0000), indicating that the upper jaw achieved better precision. In scenarios where the soft tissue was included, there was no statistically significant difference, indicating similar precision (1.70 μm, *p* = 0.5234) (Table 3). Graphical evaluation of the color-coded superimposed datasets revealed high deviations (≥ 100 μm) in the palatal regions for the UU and UL scenarios (Fig. 5).

For precision, larger differences were shown for the fully prepared dental arch (FA) and with the single abutments (SA). In the latter case, significant differences in precision were observed (4.72 μm, *p* = 0.0000). The precision of the left maxillary central incisor deteriorated significantly (2.18 μm, *p* = 0.0000) when the proximal neighbouring tooth surface was added. Further preparations in SA showed smaller but still significant increases in precision deviation when the adjacent, unprepared teeth were included.

When all preparations of FA were compared to each other, significant differences were seen in precision (*p* = 0.0139). However, no deviation tendency in relation to a specific region on the dental arch could be visually detected. When all preparations of SA were compared to each other, significant differences occurred regarding precision (*p* = 0.0008).

The bridge scenario (LB) in the first quadrant showed smaller mean deviation values (precision) (9.23 ± 0.74 μm) than the second quadrant area (16.39 ± 2.65 μm) with the proximal adjacent tooth surfaces and the ovate pontic areas included. A statistically significant difference between these two areas was calculated (7.16 μm, *p* = 0.0000). If only the preparations of both areas are compared, the difference of mean deviations was even larger (10.33 μm, *p* = 0.0000). Compared to AB (preparations only), there was a significant difference to the first quadrant (10.54 μm, *p* = 0.0000) of LB, but not to the second quadrant (0.21 μm, *p* = 0.9036). This also applies to the first (9.24 μm, *p* = 0.0000) and the second quadrant of LB (2.80 μm, *p* = 0.1017) when the adjacent, not-prepared teeth and ovate pontic areas were included in the comparison to the anterior bridge scenario. Graphical analyses revealed higher deviation values for LB in the distal region of the dental arch, especially in the second quadrant.

**Table 3:** Mean deviations (precision) in Μm, *range: minimum mean deviation (region) - maximum mean deviation (region). Tooth numbering follows the fédération dentaire internationale (FDI) system

Scan	Description	Dental arch mean ± sd	All preparations mean ± sd	Single preparations mean ± sd (range*)
UU	unprepared upper jaw with soft tissue	39.08 ± 7.03		
	- without soft tissue	29.51 ± 1.17		
UL	unprepared lower jaw with soft tissue	37.38 ± 3.03		
	- without soft tissue	39.19 ± 3.42		
FA	full arch	15.26 ± 3.41	15.26 ± 3.41	6.24 ± 0.59 (13) – 9.37 ± 3.25 (24)
SA	single abutments	22.25 ± 4.31	17.53 ± 3.04	7.54 ± 1.45 (23) – 10.69 ± 2.02 (21)
AB	anterior bridge	18.47 ± 5.27	17.70 ± 9.25	7.53 ± 0.94 (14) – 9.57 ± 1.94 (24)
LB	lateral bridges	47.70 ± 14.09	55.46 ± 16.93	6.00 ± 0.62 (16) – 14.6 ± 5.92 (26)
	− 1st quadrant		7.16 ± 0.50	6.00 ± 0.62 (16) – 7.88 ± 1.49 (14)
	− 2nd quadrant		17.49 ± 2.66	8.74 ± 2.44 (27) – 14.6 ± 5.92 (26)
PA	partials	36.0 ± 14.69	35.4 ± 16.20	7.36 ± 0.46 (26) – 14.52 ± 6.59 (24)
VE	veneers		14.97 ± 1.96	11.39 ± 1.28 (13) – 12.36 ± 2.60 (12)

**Fig. 6:**
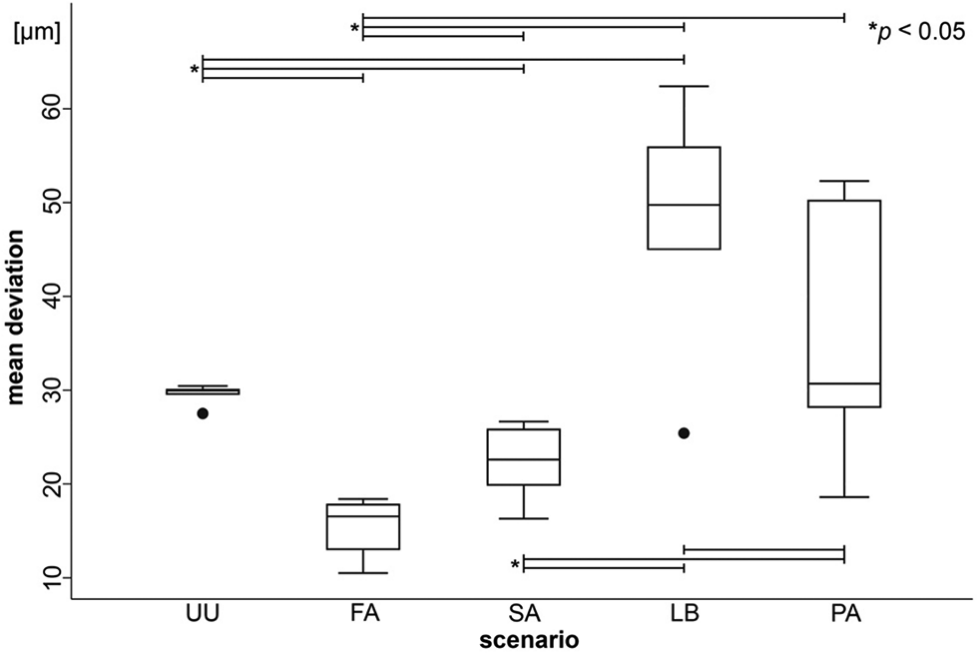
Mean deviations (precision) of maxillary arches including soft tissue. Statistically significant differences between scenarios marked with asterisks

The mean deviations (precision) of full maxillary arches are shown in Fig. 6. The comparisons between different scenarios reveal several significant findings. Significant differences in precision were observed between the unprepared upper jaw (UU) and the fully prepared dental arches (FA, SA, and LB) (*p* = 0.0000 for all), indicating that these preparation types achieved better precision compared to the unprepared upper jaw. However, the difference between UU and the partial preparations (PA) was not statistically significant (*p* = 0.0769) (Table 4).

Comparisons between fully prepared arches also showed significant differences. The full arch (FA) had significantly better precision compared to the lateral bridges (LB) (*p* = 0.0000) and the partial preparations (PA) (*p* = 0.0000). Additionally, the single abutments (SA) demonstrated significantly better precision compared to the lateral bridges (LB) (*p* = 0.0000) and the partial preparations (PA) (*p* = 0.0000). The precision comparison between FA and SA revealed a significant difference (*p* = 0.0000), indicating that the full arch preparation had better precision. Additionally, the comparison between LB and PA showed a significant difference (*p* = 0.0024), indicating that the lateral bridges had worse precision compared to the partial preparations.

**Table 4:** Overall differences (µm) and p-values of comparisons of maxillary arch including soft tissue regarding mean deviation (precision) (**UU**: unprepared upper jaw, **UL**: unprepared lower jaw, **FA**: full arch, **SA**: single abutments, **AB**: anterior Bridge, **LB**: lateral bridges, **PA**: partials, **VE**: Veneers)

	Precision	
	Overall differences (µm)	p-value
**UU** vs. **FA**	14.25	0.0000
**UU** vs. **SA**	7.26	0.0000
**UU** vs. **LB**	18.19	0.0000
**UU** vs. **PA**	6.49	0.0769
**FA** vs. **SA**	6.99	0.0000
**FA** vs. **LB**	32.44	0.0000
**FA** vs. **PA**	20.74	0.0000
**SA** vs. **LB**	25.45	0.0000
**SA** vs. **PA**	13.75	0.0000
**LB** vs. **PA**	11.70	0.0024

## Discussion

Intraoral scanners (IOS) are increasingly becoming popular in dental practices due to their ability to provide accurate digital impressions that are comparable to conventional methods. Numerous studies have demonstrated that these devices can achieve high accuracy comparable to traditional impressions (reviewed in [21]); however, only a limited number of dental scenarios have been thoroughly investigated. This study aimed to fill this gap by evaluating a wide range of scenarios, including fully dentate upper and lower jaws, full-arch preparations, single abutments, anterior and lateral bridges, partial crowns, and veneer preparations. Our results show significant variations in accuracy (trueness and precision) depending on the dental scenario.

The trueness results indicate that fully prepared dental arches (FA) and single abutments (SA) achieved the best accuracy. The mean deviations for FA and SA were significantly lower compared to the unprepared upper jaw (UU), highlighting the improved trueness when using these preparation types. Interestingly, there was no significant difference between FA and SA in trueness, suggesting that both methods provide similar levels of accuracy. However, the lateral bridge scenarios (LB) showed significantly higher deviations, particularly when compared to FA and SA, indicating that the presence of edentulous areas negatively impacts trueness. This finding aligns with previous studies that have shown the challenges in accurately capturing edentulous regions due to the lack of anatomical landmarks [22, 23].

Precision analysis revealed that fully prepared arches (FA) and single abutments (SA) again outperformed other scenarios. The upper jaw without soft tissue showed better precision compared to the lower jaw, possibly due to the more accessible scanning areas in the upper arch. Significant differences were found between FA and other scenarios, such as LB and PA, further confirming the superior precision of full-arch preparations. The inclusion of soft tissue generally increased deviation values as described previously [4], likely due to difficulties in accurately stitching the overlapping images of undifferentiated surfaces, such as soft tissue [16].

Good access of the scanner to interproximal areas may improve the accuracy of the scan significantly [13], as can be seen when comparing unprepared (UU) to prepared dental arches (FA, SA). The inclusion of adjacent unprepared teeth in the analysis areas resulted in increased deviation values for single abutments (SA), particularly in the anterior region. This suggests that the presence of neighbouring teeth can complicate the scanning process, leading to greater inaccuracies. Similar findings were observed for partial crown preparations (PA), where complex preparation designs resulted in higher deviations compared to simpler full crown preparations. It was suggested that shiny surfaces with more reflections, present in unprepared model teeth, may have a greater risk of artifacts [5]. Additionally, steep surfaces and pointed edges of the incisors could contribute to greater inaccuracies [8, 14, 19]. The superior results observed in the fully prepared (FA) and single abutment (SA) casts likely stem from the well-defined geometric features of the preparation surfaces, such as sharp edges and flat planes. These features provide the scanner with clear and distinct landmarks, which facilitate accurate stitching during the image reconstruction process. In contrast, smoother or more rounded surfaces, such as unprepared occlusal tooth areas, offer fewer characteristic points for alignment, potentially leading to increased deviations. This observation aligns with findings from previous studies, which highlight the importance of sharp, well-characterized surfaces in improving scanner accuracy and reducing stitching errors [24, 25]. It is important to consider that the geometric characteristics of partial crown and inlay preparations (PA) differ from those of full crown preparations, which may also influence the observed differences in accuracy. Partial crown and inlay preparations typically feature sharp edges designed to facilitate precise margin identification, enhancing their suitability as landmarks for intraoral scanners. While these sharp transitions can improve the scanner’s ability to define preparation boundaries, they may also introduce artifacts or outliers due to reflections and steep surface angles, as suggested in previous studies [26, 27]. This sensitivity to sharp edges could explain the higher deviations observed in PA scenarios compared to FA or SA scans.

In contrast, full crown preparations often feature smoother transitions from the axial walls to the occlusal plateau, with distinct design requirements primarily limited to the preparation margin. These rounded geometries reduce the potential for scanning artifacts and improve data consistency, potentially explaining the superior results observed in the FA and SA scenarios. The ability of intraoral scanners to handle these geometric differences highlights the critical role of preparation design in determining scan accuracy. These findings align with prior research emphasizing that preparation geometry significantly affects scanning accuracy, with well-defined surfaces improving precision and rounded features minimizing artifacts. Further investigations into how specific geometric features influence scanner performance could provide valuable insights for optimizing both preparation design and scanning strategies. This underscores the importance of careful preparation planning and scanner guidance in ensuring accurate digital impressions, particularly in scenarios with complex anatomical features.

Similar to palatal soft tissue, edentulous alveolar ridge areas can also reduce scanning accuracy [18]. It is noteworthy that the LB scenario exhibited significantly higher deviations not only when compared to FA and SA, but also when compared to UU in terms of precision. Although some interproximal areas of the preparations are better detected by the scanner, the presence of edentulous areas seems to degrade the deviation values [18]. The bridge scenario in the second quadrant, with two missing teeth, showed higher deviations than the first quadrant with only one missing tooth. This suggests a relationship between the extent of the gap and the level of deviation values: the larger the edentulous area, the greater the inaccuracy [6, 7]. This observation is likely due to errors in the stitching process. Another factor contributing to increased deviations in the posterior dental arch, especially in the second quadrant, could be horizontal distortion, which has been noted in other studies as a result of stitching process errors [5, 8, 19]. Despite all four incisors being absent in the anterior gap (AB), no significant differences were measured compared to the gap in the second quadrant (LB). This could be due to the presence of residual papillae in the AB scenario, as well as the curvature of the anterior alveolar ridge, which may facilitate better scanner orientation and thus reduce stitching errors. The inclusion of the palate in the scanning process for the unprepared upper jaw (UU) scenario allowed for a comprehensive assessment of the scanner’s performance in capturing a fully dentate arch, including the palatal region. Previous research by Schmalzl et al. (2024) has demonstrated that scanning the palate can significantly reduce arch distortion in fully dentate cases, emphasizing its importance in such scenarios [28]. In contrast, the exclusion of the palate in other maxillary scenarios focused on specific preparation designs was intended to reduce unnecessary data complexity while maintaining clinical relevance.

The analysis of partial crown preparations (PA) revealed an increase in deviation values with increasing complexity of the preparation design [15], compared to full crown preparations. Improved access of the scanner to proximal areas can also enhance accuracy in these scenarios [13]. The results for veneer preparations (VE) were noteworthy, as these scenarios exhibited the best single tooth accuracy despite the increased risk of artifacts and shadowing in anterior teeth scans. This could be attributed to the straightforward, vestibular-only preparation design and the smaller analysis area. However, further studies are needed to confirm these findings with different veneer preparation designs to enhance clinical relevance.

One of the key strengths of this study is the use of firmly polymerized teeth in the casts, which prevented any mobility resulting from screw fixation. This approach ensured higher comparability among the scenarios, providing a solid foundation for evaluating the accuracy of IOS. The controlled in vitro conditions allowed for standardized methods, minimizing the influence of external variables and enhancing the reliability of the results. Additionally, the IOS operator practiced the scanning procedure extensively before data collection, which likely improved the consistency and quality of the scans. The Atos III Triple Scan used for reference scanning employed a multi-view structured light approach, ensuring comprehensive capture of anatomical details, including interproximal undercuts, through scans performed at multiple angles. The principle of the Atos III scanner is based on active fringe projection, where structured light patterns are projected onto the object surface and captured by two CCD cameras positioned at calibrated angles relative to the projector. This design enables precise multi-view data acquisition by adjusting the lenses and camera angles, making the scanner inherently capable of capturing complex geometries. The overlapping datasets are aligned using advanced stitching algorithms that minimize deviations during data integration. Validation studies confirm that the Atos system achieves accuracy within 2–5 μm, even when combining multiple views [29, 30]. This scanning strategy minimized the risk of unrecorded regions and provided highly accurate reference models. During scanning, the casts were carefully positioned and scanned from multiple angles to ensure the complete capture of anatomical features, including interproximal undercuts, which are often challenging to capture with single-view scans. The multi-view design of the Atos scanner, combined with its high-resolution capabilities, makes it well-suited for generating reliable reference models for comparison. Although handheld scans with the CS 3600 followed a standardized path designed to capture interproximal areas effectively, any flagged missing regions were re-scanned to ensure complete data acquisition. The operator’s experience and adherence to a systematic scanning approach further contributed to the consistency and accuracy of the CS 3600 scans. Additionally, the trimming and superimposition processes ensured that comparisons focused on the same anatomical regions, reducing the potential for bias arising from differences in scanning techniques. By defining consistent regions of interest, the study ensured that any minor deviations inherent to the scanning processes did not compromise the validity of the results. Acknowledging the limitations of combining multiple datasets, we note that the Atos scanner’s advanced calibration and alignment protocols minimize the impact of stitching errors. Nevertheless, this inherent limitation of multi-view scanning systems is acknowledged and does not detract from the robustness of the study’s methodology. Future research could explore alternative or supplementary calibration steps to further refine the accuracy of reference scans in similar experimental setups.

However, the study also has several limitations. The in vitro nature of the research means that it does not fully replicate the clinical environment. Factors such as saliva, blood, and patient movements, which are present during in vivo scans, can significantly influence the accuracy of IOS. These elements may introduce artifacts and incorrect surface data, potentially leading to worse accuracy values in clinical settings compared to those obtained in the laboratory [3, 11, 12]. One further limitation of this study is the absence of a conventional workflow, such as traditional impression techniques, as a control. Including a conventional workflow could have provided an additional benchmark for evaluating the accuracy of the intraoral scanner. Future studies should consider integrating such comparisons to offer a more comprehensive evaluation of digital and traditional workflows. The findings of this study are specific to the CS 3600 intraoral scanner and may not be directly generalizable to other devices, as variations in hardware and software can influence scanner performance. Future research should consider evaluating multiple scanners to provide broader insights into the variability across different systems. Another limitation is related to the surface characteristics of the teeth. Reflections from glossy, unprepared surfaces can result in the development of artifacts [17], affecting the quality of the scans. Different image qualities between glossy unprepared surfaces and matte preparation surfaces could impact the accuracy of the measurements. While the use of experienced operators improved the results, there is still potential for further improvement with highly skilled practitioners and optimized scanner guidance techniques [1, 2, 10]. Moreover, the determination of trueness in patient scans is inherently limited due to the absence of a reference scan, which is possible in vitro but not feasible in a clinical setting. This limitation could be partially addressed by integrating reference structures, such as pre-defined fiducial markers or artificial landmarks, into the clinical workflow. These reference structures could provide additional alignment points to enhance the accuracy of superimposition and allow for more reliable trueness evaluation in clinical environments. Additionally, some authors have proposed using measuring gauges or standardized objects placed within the field of view during in vivo scanning to serve as references for accuracy assessment [31, 32]. These gauges can provide a consistent geometric baseline for evaluating deviations in patient scans, effectively addressing some of the challenges associated with the absence of an external reference. While these approaches offer promising solutions, they also introduce practical considerations, such as the additional time required to implement reference structures or measuring gauges and the potential for interference with clinical workflows. Future studies should explore the feasibility of these methods, including their integration into routine practice and their potential to improve the reliability of trueness evaluations in vivo. Such investigations could significantly enhance the applicability of intraoral scanners in complex clinical scenarios and further bridge the gap between in vitro research and real-world practice. In this study, the comparison of data sets with and without soft tissue, including the palate, was achieved by trimming the soft tissue regions from the original scans rather than conducting separate scans for each condition. This approach ensured consistency in the scanning process while allowing for a targeted assessment of the impact of soft tissue inclusion on trueness and precision. Although trimming excluded certain anatomical landmarks, such as the palate, the superimposition process relied on the remaining anatomical features to maintain valid and meaningful comparisons. This methodological choice minimized procedural variability and enabled a focused evaluation of the effect of soft tissue inclusion on the accuracy of intraoral scans. In this study, the analysis focused on mean deviations and their standard deviations as robust measures of scanner performance, rather than maximum deviations. While maximum deviations can highlight isolated instances of high error, they are highly sensitive to outliers and may not accurately reflect the typical accuracy of the system. By emphasizing mean values and variability, the results provide a more reliable and representative evaluation of the scanner’s trueness and precision under standardized conditions. In the UU and UL scenarios, we acknowledge that intraoral scanners, such as the CS 3600, may capture interproximal areas differently or more comprehensively than the Atos III Triple Scan due to their respective scanning techniques. While interproximal areas were not excluded from these comparisons, the Atos scanner’s multi-view design ensured that these areas were captured as comprehensively as possible by scanning from multiple angles. Furthermore, the best-fit algorithm employed during superimposition minimizes deviations across the datasets, although potential outliers in interproximal regions could still influence results. To address this, consistent regions of interest were trimmed, and any regions with anomalous deviations were visually inspected during the evaluation. Another limitation of this study is the use of best-fit alignment tools in mesh analysis software for calculating deviations. These tools align STL files by minimizing deviations across the entire surface, but the measured distances reflect the closest point of the superimposed mesh to the reference mesh, rather than comparing deviations from identical real points. While this method is widely used and valid for many restorative scenarios, such as crowns or bridges, it may not be ideal for cases like edentulism requiring rigid implant-supported structures, where precise point-to-point correspondence is critical. Future research should consider alternative alignment techniques to address these specific clinical requirements.

Despite limitations, in vitro studies like this one provide valuable insights and reliable results through controlled and standardized methods. They serve as a foundation for understanding trends and potential issues that can be expected in clinical practice, guiding future research and improvements in digital impression techniques.

The trueness and precision of intraoral scans reported in this study adhere to the standards outlined in ISO 5725​. However, the clinical relevance of deviations observed in the micrometer deviation range, such as those found in this study, must be critically evaluated. Current literature lacks a definitive threshold for clinically acceptable accuracy of dental impressions and intraoral scans. The marginal fit of dental restorations is often used as an indirect measure of acceptable accuracy. This fit, however, is influenced not only by the impression or scan but also by subsequent steps in the workflow, including the manufacture of the cast and the final restoration. Each step can introduce errors that accumulate, affecting the overall fit of the dental prosthesis​ and/or restoration [8]. A study on maxillary complete-arch scans suggested that deviations up to 300 μm may be considered clinically relevant​ [33]​. However, the American Dental Association (ADA) indicates that the proper fit of a fixed prosthesis should range between 25 and 40 μm (ADA No. 8, ADA 1970/71), which is significantly lower than the 300 μm threshold​ [32]​. In the present study, the trueness and precision values were well below this threshold, with the highest reported deviations being 34.99 ± 5.35 μm for unprepared upper jaws (trueness) and 55.46 ± 16.93 μm for lateral bridges (precision). Despite the fact that the deviations observed in our study are within ranges that can be considered clinically acceptable, they highlight inherent discrepancies in accuracy associated with specific scanning scenarios. Even though clinical relevance cannot be easily quantified, achieving the lowest possible deviation remains a critical objective. This study’s findings can guide practitioners in identifying which scenarios might introduce greater discrepancies. For example, scenarios involving complex preparations or extensive edentulous areas tend to show higher deviations, suggesting that these conditions are more challenging for intraoral scanning​. In conclusion, while there is no universal consensus on the exact clinical threshold for acceptable deviations in dental impressions, the values reported in this study are within ranges considered acceptable in literature. However, the variations in accuracy across different scenarios underscore the importance of striving for minimal deviations to ensure the highest quality outcomes. This insight can help clinicians make informed decisions about when to use digital versus conventional impression techniques and highlight the need for meticulous technique and potentially additional steps in more challenging scenarios to minimize discrepancies.

## Conclusions

This in vitro study evaluated the accuracy of computerized optical impression making (COIM) across various dental scenarios, including fully dentate upper and lower jaws, full-arch preparations, single abutments, anterior and lateral bridges, partial crowns, and veneer preparations. The results demonstrated that fully prepared dental arches (FA) and single abutments (SA) achieved the best accuracy in terms of both trueness and precision. Scenarios involving edentulous areas, particularly lateral bridges (LB), exhibited higher deviations, indicating reduced accuracy. The inclusion of soft tissue, especially in palatal regions, was found to increase deviation values, likely due to challenges in the stitching process. The study also highlighted the impact of surface characteristics, with unprepared, glossy teeth surfaces contributing to higher artifact risks and inaccuracies. While this study provides valuable insights into the accuracy of IOS under controlled conditions, its in vitro nature limits the direct applicability of the findings to clinical practice. Factors such as saliva, blood, and patient movements in vivo can influence the accuracy and introduce artifacts, underscoring the need for clinical validation of these results. Further, while there is no universal consensus on the exact clinical threshold for acceptable deviations in dental impressions, the values reported in this study are within ranges considered acceptable in literature.

In summary, the findings suggest that digital impressions using IOS can achieve high accuracy in various scenarios, particularly with full-arch and single abutment preparations. However, special attention is needed when scanning edentulous areas and managing soft tissue integration. These insights can help clinicians make informed decisions about the use of digital versus conventional impression techniques, ultimately improving prosthetic outcomes and patient care. Future studies should focus on validating these findings in clinical settings to enhance their practical relevance.

## Data Availability

No datasets were generated or analysed during the current study.
